# Commentary: Phylogenomics and Comparative Genomic Studies Robustly Support Division of the Genus *Mycobacterium* into an Emended Genus *Mycobacterium* and Four Novel Genera

**DOI:** 10.3389/fmicb.2018.02065

**Published:** 2018-09-06

**Authors:** Enrico Tortoli

**Affiliations:** Emerging Bacterial Pathogens Unit, IRCCS San Raffaele Scientific Institute, Milan, Italy

**Keywords:** *Mycobacterium*, phylogenetic analysis, taxonomy, genomics, classification

I have read with interest the paper (Gupta et al., 2018) supporting the reallocation of the members of the genus *Mycobacterium* within five genera. In this paper *Mycobacterium vulneris* (van Ingen et al., 2009) was regarded as an anomalous taxon as, although being phenotypically a slow grower, it apparently presented the genomic signatures of rapid growers and clustered among the latter in phylogenomic trees. As a consequence, the species was reclassified by Gupta et al. (2018) in the novel genus *Mycolicibacterium*, created to accommodate the rapidly growing species.

We have published a study concerning the genus *Mycobacterium* (Tortoli et al., 2017) in which the phylogenomic reconstruction presents a notable resemblance to the one contemporaneously reported by Gupta et al. (2018). In our study we investigated mainly genomes sequenced by us; however, for a number of species whose genomes were already present in GenBank, including *M. vulneris* (as represented by accession CCBG00000000; Croce et al., 2014), we omitted to repeat the WGS analysis and retrieved the sequences from the public repository. As we then noticed the anomalous clustering of CCBG00000000 with rapid growers, we performed a quality control check of the CCBG00000000 sequence, which proved to be more likely derived from *Mycobacterium porcinum* (a rapid grower) and incompatible with *M. vulneris*. Therefore, we sequenced the type strain of *M. vulneris*, DSM 45247^T^. The genome sequence we obtained (NCXM01000000) found its expected phylogenomic position within the slowly growing species of the *Mycobacterium avium* complex (Tortoli et al., 2017). We conclude therefore that the transfer by Gupta et al. (2018) of *M. vulneris* (van Ingen et al., 2009) to the genus *Mycolicibacterium* is inappropriate and recommend the reinstatement of the previous basonym *Mycobacterium vulneris* (van Ingen et al., 2009).

Notably, a check of the NCBI WGS sequence database with the 16S rRNA gene sequence of *M. vulneris* (EU834055; van Ingen et al., 2009) identified genome shotgun sequence data for four other strains (ACS4093, ICS2043, ACS5020, and ACS3670; accession numbers MBEH00000000, MBEG00000000, MBEF01000000, and MBDY01000000 respectively) that are clearly mislabeled as *M. vulneris* and likely belong to the species *M. porcinum*. An effect of the misleading power of genomes such as CCBG00000000 present in GenBank. Moreover, a check of all genomes retrieved from GenBank allowed us to detect six further cases of mislabeling (Tortoli et al., 2017). Turenne et al. (2001) first pointed out the necessity for quality control in public sequence repositories. The presence of mistakes such as mislabeling and misidentification at the level of whole genomes suggests that, 17 years later, the problem has become even more serious.

## Author contributions

The author confirms being the sole contributor of this work and approved it for publication.

### Conflict of interest statement

The author declares that the research was conducted in the absence of any commercial or financial relationships that could be construed as a potential conflict of interest.
